# Genomic surveillance of malaria parasites in an indigenous community in the Peruvian Amazon

**DOI:** 10.21203/rs.3.rs-3979991/v1

**Published:** 2024-02-29

**Authors:** Luis Cabrera-Sosa, Oscar Nolasco, Johanna H. Kattenberg, Carlos Fernandez-Miñope, Hugo O. Valdivia, Keare Barazorda, Silvia Arévalo de los Rios, Hugo Rodriguez-Ferrucci, Joseph M. Vinetz, Anna Rosanas-Urgell, Jean-Pierre Van geertruyden, Dionicia Gamboa, Christopher Delgado-Ratto

**Affiliations:** Laboratorio de Malaria: Parásitos y Vectores, Laboratorios de Investigación y Desarrollo, Facultad de Ciencias e Ingeniería, Universidad Peruana Cayetano Heredia; Laboratorio de Malaria: Parásitos y Vectores, Laboratorios de Investigación y Desarrollo, Facultad de Ciencias e Ingeniería, Universidad Peruana Cayetano Heredia; Department of Biomedical Sciences, Institute of Tropical Medicine; Malaria Research group (MaRch), Global Health Institute, Family Medicine and Population Health department, Faculty of Medicine, University of Antwerp; Department of Parasitology, U.S. Naval Medical Research Unit SOUTH (NAMRU SOUTH); Department of Parasitology, U.S. Naval Medical Research Unit SOUTH (NAMRU SOUTH); Laboratorio de Salud Pública de Loreto, Gerencia Regional de Salud de Loreto; Facultad de Medicina Humana, Universidad Nacional de la Amazonía Peruana; Section of Infectious Diseases, Department of Internal Medicine, Yale School of Medicine; Department of Biomedical Sciences, Institute of Tropical Medicine; Malaria Research group (MaRch), Global Health Institute, Family Medicine and Population Health department, Faculty of Medicine, University of Antwerp; Laboratorio de Malaria: Parásitos y Vectores, Laboratorios de Investigación y Desarrollo, Facultad de Ciencias e Ingeniería, Universidad Peruana Cayetano Heredia; Malaria Research group (MaRch), Global Health Institute, Family Medicine and Population Health department, Faculty of Medicine, University of Antwerp

## Abstract

Hard-to-reach communities represent Peru’s main challenge for malaria elimination, but information about transmission in these areas is scarce. Here, we assessed *Plasmodium vivax* (Pv) and *P. falciparum* (Pf) transmission dynamics, resistance markers, and Pf *hrp2/3* deletions in Nueva Jerusalén (NJ), a remote, indigenous community in the Peruvian Amazon with high population mobility.

We collected samples from November 2019 to May 2020 by active (ACD) and passive case detection (PCD) in NJ. Parasites were identified with microscopy and PCR. Then, we analyzed a representative set of positive-PCR samples (Pv = 68, Pf = 58) using highly-multiplexed deep sequencing assays (AmpliSeq) and compared NJ parasites with ones from other remote Peruvian areas using population genetics indexes.

The ACD intervention did not reduce malaria cases in the short term, and persistent malaria transmission was observed (at least one Pv infection was detected in 96% of the study days).

In Nueva Jerusalen, the Pv population had modest genetic diversity (He = 0.27). Pf population had lower diversity (He = 0.08) and presented temporal clustering, one of these clusters linked to an outbreak in February 2020. Moreover, Pv and Pf parasites from NJ exhibited variable levels of differentiation (Pv Fst = –0.52 & Pf Fst = 0.11–0.58) with parasites from other remote areas.

No artemisin resistance mutations but chloroquine (57%) and sulfadoxine-pyrimethamine (35–67%) were detected in NJ’s Pf parasites. Moreover, *pfhrp2/3* gene deletions were common (32–50% of parasites with one or both genes deleted).

The persistent Pv transmission and the detection of a Pf outbreak with parasites genetically distinct from the local ones highlight the need for tailored interventions focusing on mobility patterns and imported infections in remote areas to eliminate malaria in the Peruvian Amazon.

## Introduction

Despite the 75% reduction in malaria cases in Peru from 2018 to 2022^1^, malaria is still a public health threat in Peru. In 2023, more than 22300 cases were reported, 84% in the Loreto region. The most predominant species causing malaria in Peru are *Plasmodium vivax* (Pv, 85%) and *P. falciparum* (Pf, 15%)^2^. In 2022, the Peruvian Ministry of Health (MINSA) launched the National Malaria Elimination Program (NMEP), aiming to reduce the number of cases by 90% in 2030 compared to 2022^1^.

Currently, hard-to-reach communities represent a challenge for malaria elimination in Peru. Indigenous populations are usually settled in these remote communities, and because of geographic isolation, MINSA cannot sustain regular interventions there. Consequently, the districts with native populations account for 75% of total malaria cases in 2020^1^.

WHO recommends malaria molecular surveillance to enrich decision-making for malaria elimination, especially in regions with remote endemic areas or with limited resources^3^, because it provides information about transmission dynamics, molecular drug resistance markers, and *pfhrp2/3* deletions, among others that serve to guide formulating or adapting NMEPs’ strategies^4^.

Previous reports of malaria molecular surveillance in Peru showed different patterns between Pf and Pv transmission dynamics^5^. Pf populations showed low to moderate genetic diversity across time and space^5,6^, with some outbreaks^7–9^ and predominant lineages since the mid-2010s^10,11^. On the other hand, Pv populations are more diverse than Pf, with a variable prevalence of polyclonal infections (10–80%) and gene flow even among distant areas^5,12–14^.

Monitoring drug resistance markers is crucial as they can alert new onsets of resistance and guide treatment strategies. The presence of validated resistant haplotypes for chloroquine (CQ) and sulfadoxine–pyrimethamine (SP) in Pf isolates has increased in the last 15 years in Peru^11,15^. However, no evidence of artemisinin (ART) Pf resistant traits has been reported yet^5,11^. For Pv, resistance marker studies focused on orthologues of Pf resistance genes, with only a few validated markers in Pv. A high proportion of Pv isolates with validated SP markers has been reported in samples from 2015–20195. Mutations in *pvmdr1* and *pvcrt* have also been found, with no correlation to resistant phenotypes^5,13^.

The deletion of *pfhrp2* and *pfhrp3* genes causes false-negative results for histidine-rich protein 2 (HRP2)-based rapid diagnostic test (RDT). Peru was the first country in the world to report parasites with pfhrp2/3 deletions (2003–2007)^16^, and later reports showed the substantial increase after 2012 (up to 70%). Noteworthy, HRP2-based RDTs have never been used in Peru for routine diagnosis^10,11,17^.

Recently, we validated two targeted next-generation sequencing assays (Pf and Pv AmpliSeq Peru) for malaria molecular surveillance^11,13^. The AmpliSeq assays include country-specific SNP barcodes, molecular resistance markers, and *pfhrp2/3* genes. Using Ampliseq, our team reported Pf lineages with low genetic diversity, double *pfhrp2/3* deletion, and resistance haplotypes, except for ART, predominant after 2014^11^. In addition, we showed that Pv transmission is heterogeneous in different settings in the Peruvian Amazon, with high diversity in areas close to Loreto and lower diversity in border areas^13^. However, our molecular surveillance studies mainly focus on urban areas or accessible rural communities. Therefore, information on malaria transmission and molecular epidemiology in hard-to-reach areas with indigenous populations is scarce.

Here, we utilized the Pv & Pf AmpliSeq Peru assays to investigate malaria transmission dynamics and surveillance of molecular resistance markers and *pfhrp2/3* deletion in Nueva Jerusalen - a remote, indigenous community in the Loreto region with persistent transmission. This information highlights the importance of understanding malaria in hard-to-reach areas that can help to propose specific intervention strategies for malaria elimination in Peru.

## Materials and Methods

### Study sites and Sample collection

**Figure 1:**
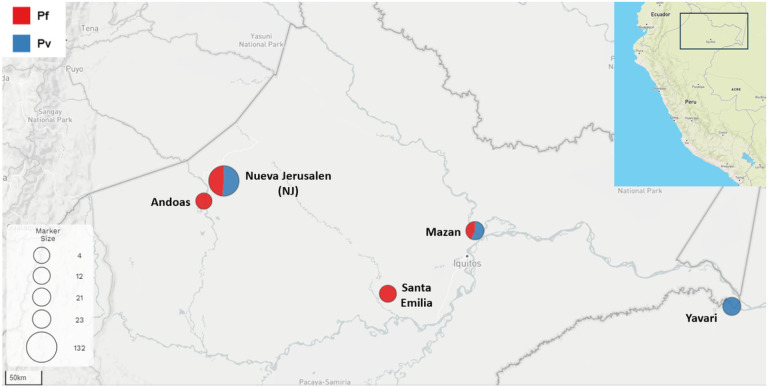
Study sites and Sample selection. The map shows the 5 areas where samples were collected. Nueva Jerusalen (NJ, in bold) was the main community in this work. The circles sizes represent the number of Pv and Pf samples used from each area.

Nueva Jerusalen (NJ) is a remote Ashuar indigenous community in the Peruvian Amazon (Fig. 1). NJ belongs to Trompeteros district, Loreto province, Loreto region (2°50’12.4 “S 76°11’29.8 “W) and it is located more than 340 km from Iquitos city, Loreto’s regional capital, and approximately 50 km from the Ecuador border. NJ has 521 inhabitants (including the annexed community Nueva Nazareth) and has a health post with a laboratory technician, an obstetrician, and a physician. Wooden houses are predominant in NJ, and the climate is hot, tropical, and humid throughout the year.

As part of MINSA’s surveillance activities, samples in NJ were collected by active (ACD) and passive (PCD) case detection. First, three consecutive ACD interventions (voluntary open call at the health post for people present in the community) were conducted at intervals of 7 days (from the end of November 2019 to the start of December 2019). In addition, PCD collection (symptomatic people attending the health post) was conducted from the first week of December 2019 to mid-May 2020. Due to logistical issues, samples were not collected in January 2020 and the first half of March 2020. Blood samples were collected by finger prick on glass slides for light microscopy detection^18^ at the local health post by well-trained microscopists following national guidelines. Samples were also collected on filter paper for later molecular diagnosis at the UPCH research laboratory in Lima. Regardless of the symptoms, treatment was provided for any microscopy-detected infection at the NJ health post, following the national guidelines^18^. Because of NJ inhabitants’ high mobility, treatment completion was not always ensured.

For comparison, we also used malaria samples previously collected in other remote areas by our team (Mazan, Santa Emilia, Andoas, and Yavari; Fig. 1). The Mazan district is located 55–60 km from Iquitos city (1h by boat through the Amazonas River) and is surrounded by Mazan and Napo rivers. Agriculture, lumber, and fishing are the main economic activities^19,20^. Samples from Mazan were collected by two population-based cross-sectional surveys in 8 communities in July and October 2018^21,22^. Santa Emilia is located about 120 km from Iquitos city. The access route is by traveling from Iquitos to Nauta city (4 h on the road) and then 144 km by fluvial displacement for 12 h (Marañon River). Although agriculture is the main economic activity, people often go to Nauta for bartering^14^. Samples from this community were collected by PCD and monthly ACD from March to May 2016. The district of Andoas (Datem del Marañon province) is located next to Trompeteros district (NJ), more than 360 km from Iquitos city; many communities of this district are surrounded by the Pastaza River. Indigenous people from Achuar and Quechua Achuar linguistic groups are settled here. Samples from Andoas were collected by ACD in September and October 2018. The district of Yavari (Mariscal Ramon Castilla province) is 364 km away from Iquitos city and is surrounded by the Yavari River. In particular, the Islandia community is part of the “Triple border” between Peru, Colombia, and Brazil. The accessway from Iquitos is fluvial (12 h, accessible every other day). Samples from Yavari were collected by PCD in December 2018.

### Ethics

Sample collection of all different projects was registered and approved by the Institutional Ethics Committee at Universidad Peruana Cayetano Heredia (UPCH). (SIDISI codes: 64024, 101518 and 102725). The participants and/or their legal guardians provided written informed consent during the study’s enrolment. In NJ, informed consent was also obtained from the Apu (community leader). Some samples were collected as part of the MINSA interventions for diagnosing and treating malaria cases and then transferred to our team for research purposes. The molecular surveillance part of this study was also approved by the Institutional Ethics Committee at UPCH (SIDISI code: 207543) and the Research Administration Program of NAMRU SOUTH (NAMRU6.2019.0008). All methods were performed following the MINSA guidelines and regulations.

### Sample processing

DNA was extracted from dried filter paper blood spots (two disks of 6 mm^2^ only for NJ or one portion of 8 ×9 mm^2^) or packed red blood cells (40ul, only Mazan) using EZNA^®^ Blood DNA (Omega Bio-tek, USA), following the manufacturer’s protocol. Elution volume was 50ul in all cases. DNA was stored at −20°C until use.

Molecular diagnosis was performed by different real-time PCR (qPCR) protocols. Samples from Mazan, Andoas, and Yaravi were diagnosed using a SYBR Green-based assay and species identification by melting temperature^23^. A species-specific TaqMan probe assay was used in samples collected in NJ in 2020 and Santa Emilia^24^. Finally, Samples from NJ in 2019 were diagnosed using a double-step protocol^25^. First, the external primers from the Rougemont assay were used in a SYBR Green-based qPCR. After that, the TaqMan assay was used only with positive infections in the first reaction.

Pv and Pf positive samples selected from the studies described above were processed again to ensure DNA quality for the sequencing. DNA was re-extracted using the EZNA^®^ Blood DNA Mini kit, and the Mangold protocol^23^ was performed. Samples with parasitemia > 5 par/μl were randomly selected by each study site. These procedures were done up to one week before the sequencing runs.

### AmpliSeq Assays

Pv and Pf AmpliSeq Peru library preparation was performed as previously described^11,13,26^ using the AmpliSeq Library PLUS kit (Illumina). Briefly, each sample (7.5 μl of DNA) was amplified by PCR using two different sets of primer panels. Then, the PCR products were mixed and partially digested with the FuPa reagent, and indexes were ligated. Next, a washing step was performed with Agencourt AMPure XP beads (Beckman Coulter). Once ready, the library was amplified by PCR and washed again to remove genomic DNA and residual primers. Subsequently, the amplified library was quantified using the Qubit High Sensitivity DNA kit (Invitrogen). Libraries from each sample were diluted to 2 nM and mixed, using equal volume for each library, to form a pool. Finally, denaturation by NaOH was performed and further diluted to a final concentration of 7 pM. PhiX was added at 1% (Pv) or 5% (Pf). The final pool was loaded onto the MiSeq for 2 × 300 cycle pair-end sequencing using the Miseq Reagent Kit v3 (Illumina).

FASTQ files generated on the MiSeq were processed using an analysis algorithm based on the Unix operating system^26^. In summary, quality control of the FASTQ files was performed with the FastQC program^27^. Then, indexes and low-quality reads were removed with Trimmomatic^28^. The trimmed reads were aligned with the reference genome (PvP01 version 46 for Pv or Pf3D7 version 44 for Pf from PlasmoDB, https://plasmodb.org/plasmo/app) with the Burrows-Wheeler aligner (BWA) program^29^. Variants were called using the Genome Analysis Toolkit (GATK) program^30^, generating a gVCF file for each sample. Individual gVCF were combined to call genotypes jointly. Then, a hard filter was performed with GATK, and the variants that passed were annotated with SnpEff^31^.

Coverage depth per locus was used to calculate the median depth for all loci per sample, per locus, or amplicon. Aligned coverage was calculated as the number of bases that passed the filters divided by the total number of bases involved in the AmpliSeq assay (59,815 bp for Pv and 57,445 bp for Pf).

### Inclusion criteria for analysis

For subsequent analyses, samples with good data quality (mean coverage > 15 reads/position, % genotype missing < 35% for Pf and < 25% for Pv) were selected. In this sense, 101/122 (82.8%) for *P. vivax* and 83/90 (92.2%) for *P. falciparum* samples were used in the AmpliSeq assays (Supplementary Table S1).

### Data analysis

The within-sample F statistic (Fws) was obtained using the moimix package to determine the complexity of the infection^32^. A monoclonal infection was considered when the Fws was ≥ 0.95. All biallelic SNPs detected by the AmpliSeq assays were included in this calculation.

Population genetics analyses provided insights into NJ’s Pv and Pf transmission dynamics. Genetic diversity was expressed as expected heterozygosity (*He*) and was calculated using the adegenet package^33^. Genetic differentiation was measured as Fst^34^ using the hierfstat package^35^. The respective AmpliSeq assays specific SNP barcodes were used for genetic diversity and differentiation.

Principal component analysis (PCA) using the prcomp function in stats R-package and discriminant analysis of principal components (DAPC)^36^ were performed with the adegenet package to assess population structure. All variants were included in PCA and DAPC analysis.

The PED and MAP format files for all variants were created using VCFtools for identity-by-descent (IBD) analysis. For this purpose, the IBD-sharing between pairs of samples was calculated using the isoRelate package^37^. Genetic distance was calculated using an estimated mean from *Plasmodium chabaudi* map unit size of 13.7 kb/centimorgan (cM)^38,39^ for Pv and 17.141 kb/cM for Pf^37,40^. For both species, the IBD threshold is established at the minimum number of SNPs (n = 10) and the length of the IBD segments (1000 bp). IBD networks shared between samples were created using the igraph package^41^.

Phylogenetic trees were built using the neighbor-joining method with the ape package^42^ using all biallelic SNPs to determine phylogenetic relationships between samples. The trees were visualized in Microreact^43^.

Lists of variants of interest in drug resistance-associated (or potentially associated) genes for Pv and Pf, originally created by literature search^11,13^, were used in this study. Haplotypes were created by combining genotypes of major variants of interest in each gene.

#### pfhrp2/3 genotyping

The presence or absence of *pfhrp2* and *pfhrp3* genes was determined by the Pf AmpliSeq assay^11^ and conventional PCR^16,44^. The PCR protocol consisted of 2 steps. First, separated PCRs amplifying *msp1, msp2,* and *glurp* genes^45^ were performed as DNA quality control. Samples that amplified at least 2 of these genes went to the second part, where exon 2 from *pfhrp2* and *pfhrp3* genes were amplified separately by PCR^44^. Primers and PCR conditions are in Supplementary Table S2. In both cases, the presence or absence of genes was determined by agarose gel electrophoresis visualization. DNA from 3D7 (*pfhrp2+, pfhrp3−*), Dd2 (*pfhrp2−, pfhrp3+*), and HB3 (*pfhrp2+, pfhrp3−*) strains were used as controls. DNA from a healthy donor and no reaction control were also included. Results from the AmpliSeq assay and PCR were then compared.

### Statistical Analysis

All statistical analyses were performed using R (version 4.2.2) and R Studio (version 2022.12.0). The Z-test or Chi-squared test was used as appropriate to compare proportions. In addition, the Mann-Whitney U test or Student’s t-test was used to compare continuous variables according to their normality. Cohen’s Kappa coefficient was used to assess the agreement between PCR and the algorithm using the AmpliSeq assay. P values < 0.05 were considered significant.

## Results

### Malaria epidemiology in NJ

From November 2019 to March 2020, 2678 samples were collected in NJ (Table 1). Considering both types of collection (ACD and PCD), 744 *Plasmodium* infections were detected by microscopy and 862 by PCR. Most infections were due to Pv (92%, 682/744 by microscopy; 89%, 771/862 by PCR). Moreover, nine mixed infections (Pf + Pv) were detected, five only by PCR and other four by microscopy.

**Figure 2:**
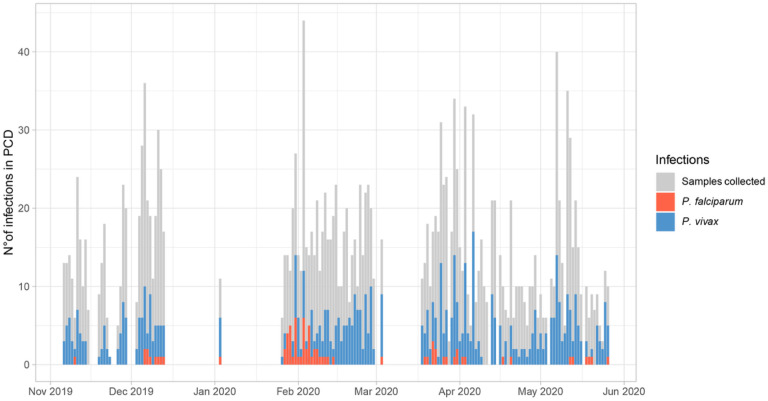
Daily distribution of infections due to *P. vivax* and *P. falciparum* during PCD in NJ. The bars represent the number of PCR-positive infections in each day.

Most infections detected by PCD were due to Pv (657/739, 88.9%) (Fig. 2). On most days (96%, 128/133 days), at least one infection was detected by PCR. Overall, the median number of Pv infections per day was 4 (IQR: 2–6), indicating a persistent Pv malaria transmission in the community. In contrast, Pf infections displayed temporal patterns, particularly peaking in February and March (Fig. 2).

### Effect of ACD intervention in NJ

**Figure 3:**
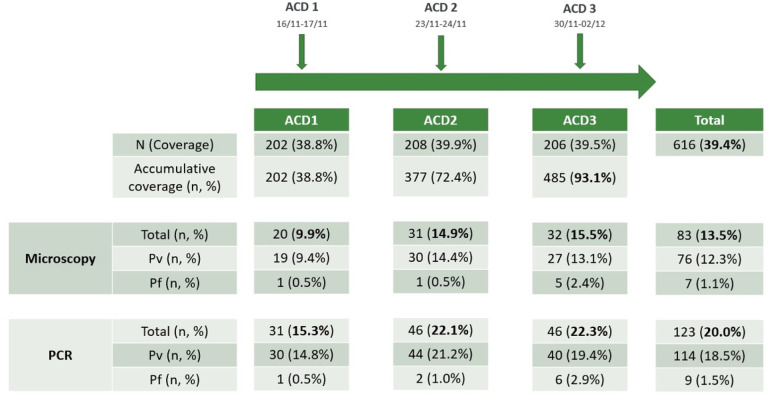
Effect of ACD intervention on malaria in NJ. The positive rate in each weekly ACD visit, determined by microscopy or PCR, on malaria infections and by species is shown.

Three active case detection (ACD) visits were conducted at intervals of 7 days. To describe the short-term effect of ACD on reducing malaria infections in NJ, the changes in the malaria positivity rate by microscopy and PCR during those visits were calculated (Fig. 3).

Out of the 521 inhabitants, the mean coverage in the weekly ACD was 39.4%. However, cumulative coverage (the proportion of the population with at least one sample collected) was 93% after the three interventions. Overall, 83/XX (13.4%) and 123/XX (20%) malaria infections were detected by microscopy and PCR, respectively.

The positivity rate in each ACD varied between 10 and 15% by microscopy and 15 to 22% by PCR. The ACD 1 (n = 202) had the lowest positivity rate by microscopy (9.9%) and by PCR (15.3%) compared to the other ACDs (ACD 2: n = 206, 14.9 and 22.1%; ACD 3: n = 208, 15.5 and 22.3%). There was no difference in the positivity rate by microscopy or PCR among the three visits.

Approximately 20% of the inhabitants (101/521) had samples collected in more than one ACD visit. A third of them (34%, 34/101) had at least one positive PCR result. On the other hand, nine individuals had a positive PCR sample 1–2 weeks after a negative PCR sample.

### Pv microepidemiology in NJ

To investigate temporal changes in Pv population structure in NJ, we analyzed samples collected on each ACD visit from Nov-Dec 2019 (ACD 2019) and from April and May 2020 (PCD 2020) with the Pv AmpliSeq assay (Supplementary Table S1). Polyclonal Pv infections accounted for 32 to 46% (Supplementary Table S3), without differences across time (p = 0.83).

**Figure 4:**
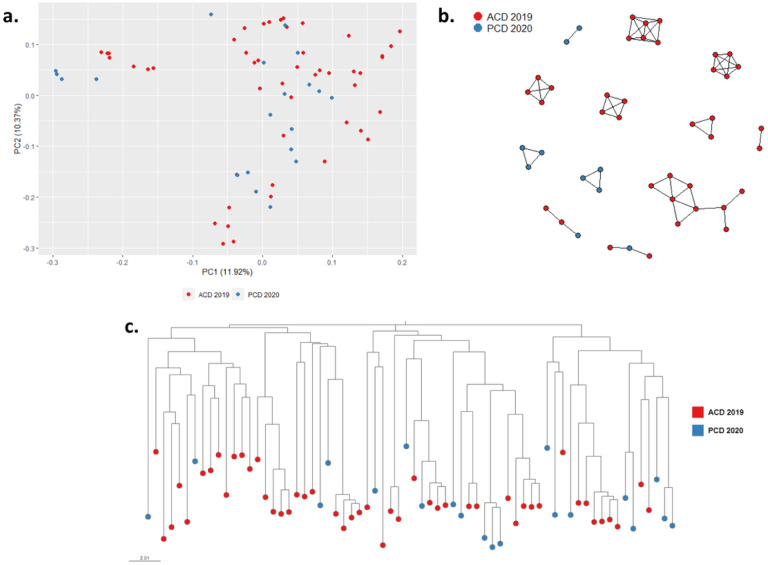
Population structure and parasite connectivity of Pv in NJ. (a) PCA of 68 Pv samples in NJ. (b) Network inferred by IBD between *P. vivax* isolates from NJ. Edges connecting parasite pairs indicate that >45% of their genomes descended from a common ancestor. (c) Neighbor-joining network of *P. vivax* samples. All analyses showed the absence of temporal clustering.

No temporal clustering was detected in ACD 2019 and PCD 2020 (PCA, Fig. 4A), neither among the ADC visits (Supplementary Fig. S1). Pv population exhibited modest level of genetic diversity (*He* = 0.35–0.38) and low genetic differentiation (Fst = 0.01–0.1) between collections across time (Supplementary Fig. S1).

A network of inferred IBD between Pv sample pairs was generated to evaluate connectivity within the community (Fig. 4B). The Pv population in NJ featured multiple genetic clusters. However, no cluster was exclusive for a specific period, i.e., some clusters were present throughout the study period, indicating the presence of multiple local haplotypes circulating in NJ during the study period. A similar result was obtained with the phylogenetic analysis (Fig. 4C).

### Pf microepidemiology in NJ

To explore differences between the parasites observed in temporal patterns of Pf infections shown in Fig. 2, we analyzed Pf samples collected in November and December 2019 and February, March, and April-May 2020 (Supplementary Table S1). Pf polyclonal infections accounted for 21% in 2019 and 39% in 2020 of overall infections (Supplementary Table S3). The proportion of polyclonal infections was higher in Feb 2020 (58.6%) compared to the other months (p < 0.001).

**Figure 5:**
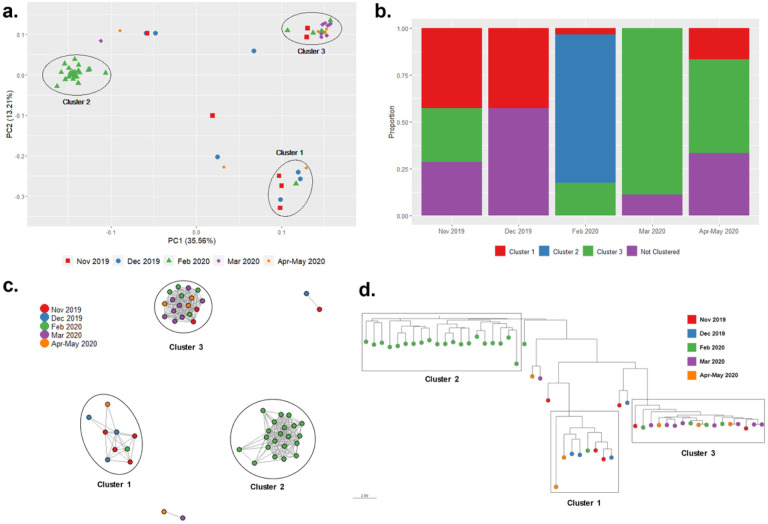
Population structure and parasite connectivity of Pf in NJ. (a) PCA of 58 Pf samples in NJ. The shape/color schemes represent to each month. (b) Relative proportion of each cluster in the different months. (c) Network inferred by IBD between Pf isolates from NJ. Edges connecting parasite pairs indicate that >45% of their genomes descended from a common ancestor. (d) Neighbor-joining network of Pf samples. All analyses showed sub-structuring in 3 clusters (depicted by ellipses), highlighting Cluster 2 with only samples from Feb 2020.

The Pf population was separated in 3 genetic clusters (PCA, Fig. 5A), each one composed of parasites collected in different months (Fig. 5B, Supplementary Table S4). Cluster 1 accounted for of the 43% (6/14) of samples collected in 2019 with two samples from Feb and Apr-May 2020. Cluster 2 mainly consisted of samples collected in Feb 2020 (23/29, 79.3%). Finally, Cluster 3 included most of the samples from March 2020 (8/9, 88.9%) and some samples from other periods (February to May 2020). In the PCA, Cluster 2 was clearly separated from the other clusters, which was also supported by the DAPC analysis (Supplementary Fig. S2). The temporal clustering was also observed in the IBD network (Fig. 5C) and the phylogenetic tree (Fig. 5D).

Genetic diversity in the Pf population was low (*He* = 0–0.2). Pf parasites from Cluster 3 (He = 0) were less diverse than parasites from Cluster 2 (He = 0.08, p = 0.008) (Supplementary Fig. S2). Moreover, high genetic differentiation was noted among the clusters (Fst = 0.44–0.85) where Cluster 3 was the most differentiated (Fst = 0.59–0.85) (Supplementary Fig. S2).

### Comparison between NJ and other remote areas

Regardless of the time of collection, NJ Pv parasites were slightly differentiated from Mazan parasites (Fst = 0.07–0.11), which in turn had modest diversity (He = 0.35). NJ parasites were highly differentiated from Yavari parasites (Fst = 0.43–0.52), which had a clonal population (He = 0.01) (Supplementary Fig. S3).

**Figure 6:**
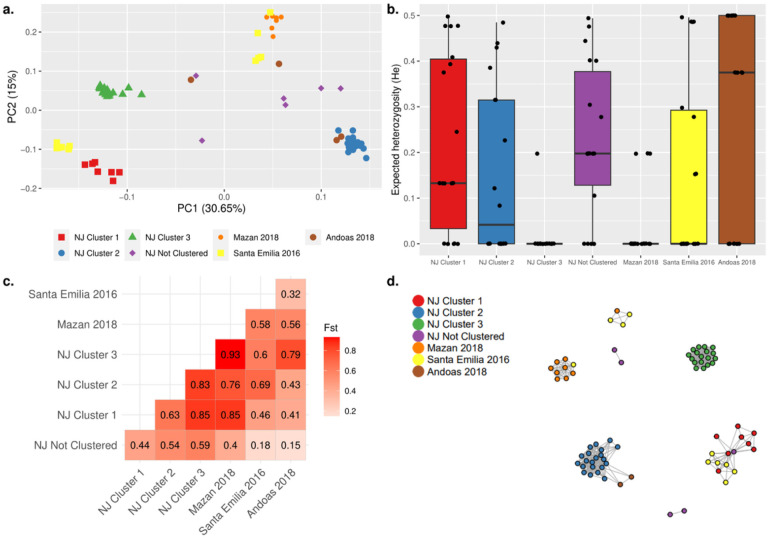
Population genetic analysis and connectivity of Pf samples from NJ (n = 58) and other remotes areas: Mazan (n = 9), Santa Emilia (n = 12), Andoas (n = 4). (a) PCA of Pf samples, showing some clusters with samples from different areas. The shape/color schemes represent to each of area/time of collection. PCA. (b) Expected heterozygosity (He). Each dot represents the mean He of 17/28 non-fixed positions from the SNP barcode for all samples in each group. Low to moderate diversity was noted. (c) Pairwise Fst statistic among the groups. The heatmap color scheme was based on the maximum and minimum of Fst values (numbers at the center of each square). Mazan samples were the most differentiated. (d) Network inferred by IBD between *P. falciparum* isolates. Edges connecting parasite pairs indicate that >45% of their genomes descended from a common ancestor. Node colors indicate the 5 groups. Clustering pattern was similar to PCA.

Distinct clusters of Pf parasites from different districts in Peru were observed (Fig. 6). NJ Pf parasites from Clusters 1 and 2 were clustered with some parasites from Santa Emilia and Andoas, respectively. In addition, a cluster composed of parasites from Mazan and Santa Emilia was also observed (Fig. 6A). Also, Pf parasites from NJ Cluster 1 (He = 0.13, p = 0.003) and from Andoas (He = 0.38, p = 0.002) were more diverse than Mazan parasites, which were observed a clonal linage (Fig. 6B). Modest to high pairwise genetic differentiation (Fst = 0.32–0.93) among the 3 NJ clusters and parasites from other areas was noted (Fig. 6C). The connectivity pattern in the IBD network (Fig. 6D) showed similar clustering as in the PCA.

### Drug Resistance Markers in NJ and other remote sites

We genotyped Pf validated genes associated with resistance to different antimalarials in NJ and other remote areas in the Peruvian Amazon using the Pf AmpliSeq assay (Supplementary Table S5). For *pfdhfr* (pyrimethamine resistance), the triple mutation haplotype RICNI was the most common in NJ (97–100%), with 74% of parasites in NJ Cluster 2 had mixed haplotype (polyclonal, resistant + wild type). The RICNI had 100% prevalence in the other study areas. For *pfdhps* (sulfadoxine resistance), wild-type (SAKKA, 50–52%), and mixed (48–50%) haplotypes were common in NJ Clusters 1 and 2. In contrast, triple-mutation (SGEGA, 78%) haplotype was predominant in Cluster 3. No wild-type haplotype was detected. In other areas, SGEGA haplotype was the most frequent (50–92%). Wild-type haplotype was also found in Andoas (n = 2 out of 4 isolates).

The triple-mutation haplotype NDFCDY in *pfmdr1* (CQ and mefloquine resistance) was the most common in the 3 NJ clusters (87–100%) and other remote areas (78–100%). Similarly, the SVMNT haplotype in *pfcrt*, associated to CQ resistance, was very common in NJ (38–78%) and the other areas (67–100%). However, *pfcrt* was not amplified in 43% of NJ samples and 16% of samples from other sites.

Validated mutations for ART resistance in *pfk13* (F446I, N458Y, M476I, Y493H, R539T, I543T, P553L, R561H, P574L, C580YC580Y) were not detected. The K189T mutation (outside the propeller region) was predominant in NJ Clusters 1 and 2 (75–100%), meanwhile only wild-type parasites (carrying K189) were found in Cluster 3. This mutation was also frequent in Mazan (90%) and Andoas (100%), while wild-type samples were common in Santa Emilia (75%).

Similarly, mutations in *coronin* (G50E, R100K, E107V) associated with artemisinin resistance were not found. Previously reported mutations in Peru, V62M, and V424I, were also assessed. In NJ, samples carrying mixed haplotypes (including V62M) were found only in Cluster 2 (52%), while the other clusters only had wild-type parasites. The V424I mutation was only found in NJ Cluster 3, while wild-type parasites in this position were only found in the Clusters 1 and 2. In other areas, Mazan parasites had predominantly V424I (89%), but the rest of the places mainly reported wild-type samples for both 62 (100%) and 424 (75–92%) positions.

We also found previously reported haplotypes in the *ubp1* gene^11^. In NJ, the R1133S + E1011K variant was the most predominant (88–94%) in Cluster 1 and 3, while Cluster 2 only had the Q107L and/or K1193T variant. In the rest of the areas, the quadruple-mutation haplotype (R1133S + E1011K + K764N + In NJ) was present in Mazan, Santa Emilia and Andoas (33–89%).

For Pv, the genes *pvdhfr, pvdhps, pvmdr1,* and *pvcrt* were assessed (Supplementary Table S6). The FRTS haplotype from *pvdhfr*, associated to pyrimethamine resistance, was the most predominant in NJ (85% in 2019, 70% in 2020) and Mazan (46%), meanwhile FKTS was the only observed haplotype in Yavari (70). For *pvdhps*, no parasites had the A553G mutation. In contrast, the A338G mutation, associated to sulfadoxine resistance, was present in all areas (33–54%) except Yavari. For *pvmdr1,* LMYFF haplotype was the most common in NJ (77% in 2019, 90% in 2020) and Mazan (69%). However, the MMYFF haplotype was detected in all Yavari samples and Mazan (15%). Finally, only one sample in NJ had parasites with an intronic variant (357 + 83G > A) for pvcrt.

#### pfhrp2/3 genotyping

The Pf AmpliSeq assay targets the *pfhrp2* and *pfhrp3* genes, and with the difference in read depth compared to the other amplicons in the assay, the deletion can be classified. However, read depths were not consistent low or high for all 5 or 6 amplicons targeting the *pfhrp2* and *pfhrp3* genes (Supplementary Figure S4). This led to inconclusive results with the applied analysis method in 37% (31/83) and 5% (4/83) of *pfhrp2* and *pfhrp3* genotypes, respectively.

Therefore, we applied conventional PCRs targeting the exon 2 of both genes, commonly used for *pfhrp2/3* genotyping in Peru, to our samples. For *pfhrp2,* PCR detected the deletion in 49% (41/83) of all samples, while the AmpliSeq assay determined that 61% (51/83) of samples had the gene deleted (Table 2). Cohen’s kappa coefficient showed no agreement between both methods (κ=−0.038 ± 0.037) for *pfhrp2* genotyping and moderate agreement (κ = 0.493 ± 0.109) for *pfhrp3* genotyping. Most of the inconclusive samples for *hrp2* by AmpliSeq (24/31, 77%) were classified as having the gene present by PCR (Table 2).

## Discussion

Remote native communities represent a challenge for the malaria elimination strategies currently deployed in the Peruvian Amazon region^1^. In this study, we provided insights on the microepidemiology, resistance markers, and *pfhrp2/3* gene deletion in NJ. To our knowledge, this is the first report of malaria genomic surveillance in a native community in this region.

NJ has an exceptionally high malaria prevalence and persistence, exceeding previous reports in other endemic communities in the Peruvian Amazon^46^. Despite continuous diagnosis, the number of malaria cases remained unchanged during the study period. This result contrasts with a previous report in Mazan, where four consecutive ACD visits quickly reduced the disease’s prevalence^20^.

Population genetics analysis showed that the Pv population in NJ had moderate diversity, with a high proportion of polyclonal infections, without differences between 2019 and 2020. Several Pv lineages were present in NJ during the study period, commonly observed in high-transmission populations, favoring genetic recombination and increasing genetic diversity^47,48^. Similar to NJ, previous reports featured the Pv population in Peru as heterogeneous, with moderate to high levels of genetic diversity and a high proportion of polyclonal infections^12–14^, although a recent report showed a monoclonal population with low diversity in Iquitos and Mazan^5^.

The MINSA intervention in 2019 did not reduce the Pv burden in NJ. Only the ACD visits failed to decrease the cases and did not affect complexity of infection. We list here some hypotheses related to this observation, i) most adults in NJ travel out of the community for field work or social activities to nearby areas, ii) at the same time, children and their caregivers stay in the community, reflected in the low coverage in each ACD visit (Fig. 3), iii) the continuous mobility negatively affected treatment compliance and increases the likelihood of parasite importation into NJ, which can enrich the genetic diversity and the persistence of the disease in remote communities, as previously reported for the communities of the Alto Juruá river in Brazil^49^.

The transmission dynamics of Pf in NJ were different compared to Pv. The analysis showed a population substructure with 3 clusters reflecting temporal changes. Cluster 2 (February 2020) clearly differentiated from parasites detected earlier and later in the community. In addition, the IBD network showed that some parasites from this cluster were related to parasites in Andoas, a city close to NJ. This suggests that this cluster corresponds to a Pf outbreak that appeared in February 2020 in NJ. We hypothesize that new parasites were introduced (potentially from Andoas or the vicinity and were not related to local NJ parasites), rapidly spread in NJ (increase of samples collected by PCD at that time, high proportion of polyclonal infections), and then were successfully controlled (not related with later parasites). We cannot further assess the hypothesis as we lack mobility data. Nevertheless, Pf outbreaks related to similar introduction events have been reported in non-endemic regions in Peru using molecular surveillance^7,8^, and in an indigenous area in Amazonas, a Peruvian region next to Loreto, using epidemiologic data^9^.

On the other hand, Cluster 3 (March to May 2020) showed low diversity, high differentiation to other clusters, and unique features compared to the other clusters. Following our hypothesis, controlling the outbreak in Feb 2020 generated a bottleneck event in the Pf population in NJ, like those previously reported in Colombia and Honduras-Nicaragua^50,51^. Expert microscopists were present at the health post during the study period, improving diagnosis performance in the community, followed by treatment administration following national guidelines. Opportune diagnosis and treatment shut down this local outbreak.

Given NJ’s heterogeneous Pf and Pv transmission dynamics, tailored strategies should be proposed to control malaria in this scenario. In addition to better educational campaigns and vector control measures, the focus should be on working adults (the mobile population) in NJ, which must be diagnosed and treated promptly when returning to the community. This would prevent the potential spread of new parasite populations and reduce malaria transmission in the community. Malaria treatment lasts 3–7 days in Peru^18^, which may represent a problem for monitoring treatment compliance in mobile populations. Considering the limited availability of expert microscopists in remote areas, administering one standard treatment for any malaria infection is an option^52^. A single-dose tafenoquine scheme, along with glucose-6-phosphate dehydrogenase (G6PD) activity monitoring, has been tested recently in Peru and other countries and showed efficacy for the radical cure of Pv malaria^53,54^ and can be potentially used in the future. In addition, similar studies should work in testing new single-dose treatments for Pf^55^.

MINSA has implemented the training of community health agents (CHAs), people chosen by their community to develop disease prevention actions and support for the diagnosis and treatment, into the current NMEP^1^. Similar approaches have been used to control malaria in the mobile population in Myanmar^56^ and in an indigenous area in Panama^57^, showing promising results in each case. In Peru, CHAs better understand the dynamics of human mobilization, facilitating the work with the population. However, an adequate number of CHAs, enough resources, and continuous monitoring of their work are necessary to ensure the success of this strategy to control malaria. In addition, due to the geographical location and the socio-cultural context, the participation of the different stakeholders is also crucial^58^. We also recommend conducting qualitative studies in indigenous areas to understand their point of view about malaria and their mobility behavior^59^ for a better strategies adaptation.

For Pf, the most frequent haplotypes were associated with drug resistance: RICNI (*pfdhfr*) and SGEGA (*pfdhps*) to SP^60^, NDFCDY (*pfmdr1*) and SVMNT (*pfcrt*) to CQ^61,62^. An increase in the frequency of these haplotypes has been reported in recent years^5,11^. Fortunately, none of these are drugs used in the current treatment scheme against Pf in Peru^18^. In this regard, no validated mutation for artemisinin resistance has been found in this study, being concordant with previous studies^5,11,63^.

The Pv AmpliSeq assay includes putative resistance-associated markers. Most correspond to orthologs to genes in Pf associated with resistance^13^. Only two markers are validated for Pv: *pvdhps* (positions 383, 553) and *pvdhfr* (positions 57, 58, 61, 117, 173) for SP resistance^64^. The A553G mutation in *pvdhps* and the S58R, S117N, and I173L mutations in *pvdhfr* were present in 33–95% of the samples in NJ and Mazan, comparable to a previous report^5^. The presence of these mutations in Pv may be explained by exposure to SP due to mixed infections or missing diagnosis^65^, although SP ceased to be used against Pf more than 20 years ago^66^. In the case of *pvmdr1*, the LMYF haplotype was the most abundant (69–77%) in NJ and Mazan, which is consistent with previous surveillance reports on this gene^5,67^.

The first report of the deletion in the *pfhrp2/3* genes and its relationship to failure in rapid diagnostic tests was in Peru^16^. Since that report, the frequency of these deletions in the country has increased after 2012 up to 70%^10,11,17^. In contrast, the moderate proportion of samples with *pfhrp2/3* genes (50–67%) showed that deletions have not yet spread to hard-to-reach communities such as NJ. In our study, the double deletion was only predominant in Mazan.

In this study, we found different results in *pfhrp2/3* genotyping between PCR and the Pf AmpliSeq assay. There are also some differences in the regions targeted by these two assays that could, for a large part, explain these contrasting results. The Pf AmpliSeq assay targets the genes with several amplicons that together span the full length of the genes. In the analysis applied here, we determined a ratio of the depth of the *pfhrp2* or *pfhrp3* amplicons compared to all other amplicons targeted in the assay, and a deletion is determined when multiple amplicons have decreased depth^11^. When amplicons for one gene yield contrasting results (which could occur when only part of the gene is deleted), an inconclusive result is generated. The PCR used in this work only targets only exon 2 of each gene in a conventional PCR protocol^16,44^.

In addition, there are reports that showed different breakpoints for the deletions in both genes, that can impact the accuracy of both analysis approaches applied here^68^. In the case of *pfhrp2*, the breakpoint often encountered in Peru are in exon 2 or the neighboring intron, and therefore partial deletion of *pfhrp2* gene is expected, especially deletion of exon 1. On the other hand, for *pfhrp3*, the deletion in Peru is explained by a recombination with chromosome 11, thus a deletion of the entire gene is expected. Further work is needed to structurally characterize *pfhrp2/3* deletions in Peru, particularly in remote areas not previously included in studies characterizing these genes. Here, we have detected different Pf clusters than those from previously studied areas^5,11^. Using updated structural variation information from more in depth studies in these regions, we can re-evaluate and optimize the approach of which amplicons to use in the determination of deletions based on the read depths in the Pf AmpliSeq assay. Besides that, homology, repetitiveness, and high %AT sequences of both genes could have complicated alignment of reads in certain regions of the genes.

This work has some limitations. Initially, we planned to carry out a treatment efficacy study in the community, following a cohort for two years. At the beginning, samples were collected as part of MINSA interventions, and no additional epidemiological data were collected. Unfortunately, all work stopped due to the COVID-19 pandemic. Second, we used sampling by convenience, leading to the analysis of different sample sizes by place and samples with different collection methods and times. Because of high sequencing costs^11^, we only performed one run per species assay. Today, collaborating with international consortia (such as MalariaGEN) for sequencing is more common in countries with high costs and scarce infrastructure. In addition, the heterogeneous malaria transmission in Peru must be considered to make molecular surveillance representative of the country^11,13^. Moreover, the *pfcrt* gene could not be genotyped in 35% of samples. Since in previous studies, this gene performed well, we should investigate the genetic variability in the primer regions of the amplicons.

In conclusion, NJ, an indigenous remote community in the Peruvian Amazon with high transmission and persistent malaria, has local Pv parasites with modest genetic diversity. In contrast, the Pf population has low genetic diversity and temporal clustering. An outbreak represented by a unique genetic cluster was detected in February 2020, which was controlled through timely microscopy diagnosis by expert personnel and treatment administration. The Pf population carried mutations associated with CQ and SP resistance but not with artemisinin. Additionally, the presence of *pfhrp2* and *pfhrp3* genes was frequent in NJ and other remote areas, except in Mazan, where the double deletion predominated.

This information highlights the importance of regular molecular surveillance in indigenous remote communities, preferably linked to MINSA activities, regarding imported cases, transmission dynamics, and spread of resistance, to promptly adapt malaria elimination strategies in Peru. In this regard, we showed that AmpliSeq assays, with some improvisations, can be helpful for NMEP.

## Figures and Tables

**Figure 7:**
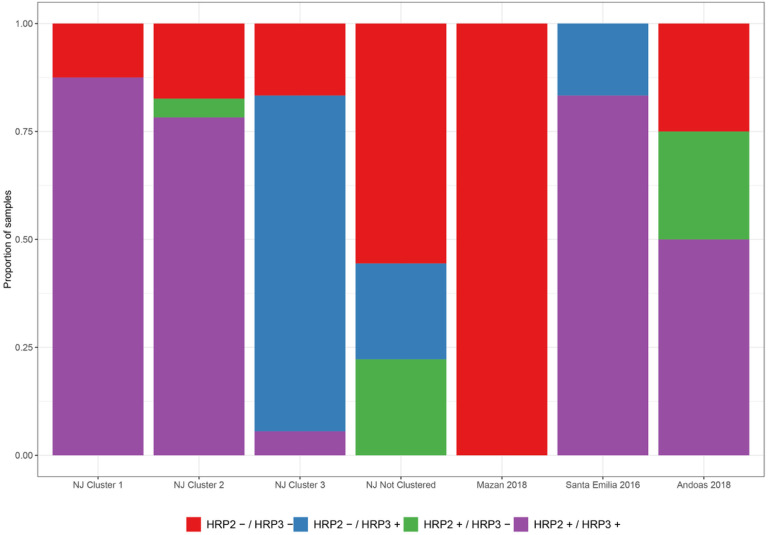
pfhrp2 and pfhrp3 genotyping. PCR results were used to create the pfhrp2/3 genotypes in all areas (A) or within NJ (B). Double deletion was predominant in Mazan, but both genes were present in the rest of areas. In NJ, double deletion, *pfhrp2+ / pfhrp3+* and *pfhrp2− / pfhrp3+* was common in 2019, February 2020 and March to May 2020, respectively.

**Table 1:** Malaria infections during active (ACD) and passive (PCD) case detection in Nueva Jerusalen

	ACD 2019 (n = 616)	PCD 2019 (n = 468)	PCD 2020 (n = 1594)
***Plasmodium***			
Microscopy (n, %)	83 (13.5%)	107 (22.9%)	554 (34.8%)
PCR (n, %)	123 (20.0%)	139 (29.7%)	600 (37.6%)
PCR Parasitaemia [par/ul, Me (IQR)]	55.2 (8.4–570)	368 (16.3–1616)	777 (95–3928)
**P. vivax**			
Microscopy (n, %)	76 (12.3%)	102 (21.8%)	504 (31.6%)
PCR (n, %)	114 (18.5%)	127 (27.1%)	530 (33.2%)
PCR Parasitaemia [par/ul, Me (IQR)]	49.5 (7.9–410)	383 (17.6–1782)	715 (84.9–3245)
**P. falciparum**			
Microscopy (n, %)	7 (1.14%)	5 (1.07%)	46 (2.88%)
PCR (n, %)	9 (1.46%)	10 (2.14%)	67 (4.20%)
PCR Parasitaemia [par/ul, Me (IQR)]	732 (81.5–6893)	90 (10.2–755)	6794 (267–14732)

Me: median, IQR: interquartile range

During 2019, the proportion of infections detected in PCD was higher than in ACD regardless of the diagnostic method (23 vs. 13.5% by microscopy ; 30 vs 20% by PCR, p < 0.0001 for both). In addition, the proportion of infections detected in PCD in 2020 was higher than in 2019 (35 vs 23% by microscopy; 38 vs 30% by PCR, p < 0.002 for both).

**Table 2:** Comparison of PCR and AmpliSeq assay results for *pfhrp2/3* genotyping

*pfhrp2*		AmpliSeq			
		Deletion	Presence	Inconclusive	Total
**PCR**	Deletion	33	1	7	41 (49.4%)
	Presence	18	0	24	42 (51.6%)
	**Total**	51 (61.4%)	1 (1.2%)	31 (37.3%)	83 (100%)
pfhrp3		AmpliSeq			
		Deletion	Presence	Inconclusive	Total
**PCR**	Deletion	10	13	4	27 (32.5%)
	Presence	1	55	0	56 (67.5%)
	**Total**	11 (13.3%)	68 (81.9%)	4 (4.8%)	83 (100%)

Using the PCR data for *pfhrp2/3* genotyping, which was able to genotype all samples, parasites from NJ Clusters 1 and 2 were predominantly genotyped as pfhrp2+ / pfhrp3+ (87% and 78% respectively), while pfhrp2− / pfhrp3 + was common in Cluster 3 (78%; Fig. 7, Supplementary Table S7). Parasites with both genes present were frequent in Santa Emilia (83%) and Andoas (50%). However, all parasites from Mazan carried the double deletion of both genes.

## Data Availability

Sample metadata, drug resistance, and *pfhrp2* and *pfhrp3* haplotypes, locations, and dates are accessible in the Supplementary Files 1 (Pv) and 2 (Pf). In particular, information about NJ Pf clusters’ drug resistance markers and *pfhrp2/3* genotypes is accessible at https://microreact.org/project/12yFxakVcYwNT1JjFC8H1K-nj-pf-clusters. Raw data (FASTQ files) are available at the SRA under BioProject accession numbers PRJNA1055117 (Pv) and PRJNA1074830 (Pf). Individual library accession numbers are listed in the Supplementary Files 1 and 2. Variant files (vcf) and scripts are available upon request. A Spanish version of the main manuscript is available in Supplementary File 3.
